# Clinical efficacy of weekly cisplatin for treatment of patients with breast cancer

**DOI:** 10.1097/MD.0000000000017114

**Published:** 2019-09-13

**Authors:** Ying Ma, Nai-peng Zhang, Ning An, Wen-yuan Li, Wei Zhao, Yan-cui Liu

**Affiliations:** a Department of Library, Mudanjiang Medical University; b Department of Urology; c Second Ward of Neurology Department, Affiliated Hongqi Hospital; d Institute of Neural Tissue Engineering; e Department of Anatomy, Mudanjiang Medical University, Mudanjiang, China.

**Keywords:** breast cancer, efficacy, safety, weekly cisplatin

## Abstract

**Background:**

We will investigate the efficacy and safety of weekly cisplatin (WC) for treatment of patients with breast cancer (BC) systematically.

**Methods:**

This study will describe and critically appraise shared decision approaches used in randomized controlled trials of WC for treatment of patients with BC. We will comprehensively search the following databases: PubMed, EMBASE, Web of Science, Cochrane Library, CINAHL, PsycINFO, Allied and Complementary Medicine Database, Wanfang, and Chinese Biomedical Literature Database from inception through July 1, 2019. We will utilize RevMan V.5.3 software (London, UK) for statistical analysis.

**Results:**

This study will systematically explore the efficacy and safety of WC for the treatment of patients with BC through evaluating primary outcomes of overall survival, pathological complete response; and secondary outcomes of cancer-specific survival, recurrence-free survival, disease-free survival, quality of life, and toxicities.

**Conclusion:**

This study will provide latest evidence of WC for the treatment of patients with BC.

**Systematic review registration:**

PROSPERO CRD42019145358.

## 1 Introduction

Breast cancer (BC) is one of the most leading cancers with a high mortality rate.^[1–3]^ Among the 36 most common cancers, BC accounts for 11.6% of all of them.^[4–7]^ It has been reported that BC leads to 6.6% of cancer death in 2018.^[8,9]^ In China, it has been estimated that the rate of BC has increased and associated with the economic status.^[10]^ It has also reported that the incidence rates are 0.034% and 0.017% for urban and rural areas, respectively.^[10]^ In the United States, the cases of BC will reach 268,600 and more than 41,000 patients are predicted to die in 2019.^[9]^ In addition, there are about 3.1 million patients suffering from BC and are receiving treatments.^[10]^

A variety of clinical studies have reported that weekly cisplatin (WC) has been widely used for the treatment of patients with BC.^[11–21]^ However, its conclusion is still unclear, and no systematic review has assessed the efficacy and safety of WC for BC. Therefore, this study will systematically evaluate the efficacy and safety of WC for the treatment of patients with BC.

## 2 Methods

### 2.1 Ethics and dissemination

This study will not inquire ethic approval because we will not use individual patient data. The results of this study are expected to be published at peer-reviewed journals.

### 2.2 Eligibility criteria for study selection

#### 2.2.1 Type of studies

All randomized controlled trials (RCTs) will be selected if they focus on assessing the efficacy and safety of WC for the treatment of patients with BC. However, we will exclude studies of nonclinical studies, and non-RCTs.

#### 2.2.2 Type of participants

Any patients diagnosed with BC will be included regardless the race, age, gender, and economic status.

#### 2.2.3 Type of interventions

Experimental group: patients received WC monotherapy will be used for inclusion.

Control group: patients received any interventions except any forms of WC will be included.

#### 2.2.4 Type of outcome measurements

Primary outcomes include overall survival, and pathological complete response.

Secondary outcomes consist of cancer-specific survival; recurrence-free survival; disease-free survival; quality of life, as measured by any related scales; and toxicities.

### 2.3 Data sources and search strategy

The plan is based on a comprehensively approach to RCTs identification using the following databases: PubMed, EMBASE, Web of Science, Cochrane Library, CINAHL, PsycINFO, Allied and Complementary Medicine Database, Wanfang, and Chinese Biomedical Literature Database from inception through July 1, 2019. Additionally, we will also search dissertations, conference proceedings, and reference lists of included studies. The search strategy for PubMed is showed in Table 1.

**Table 1:**
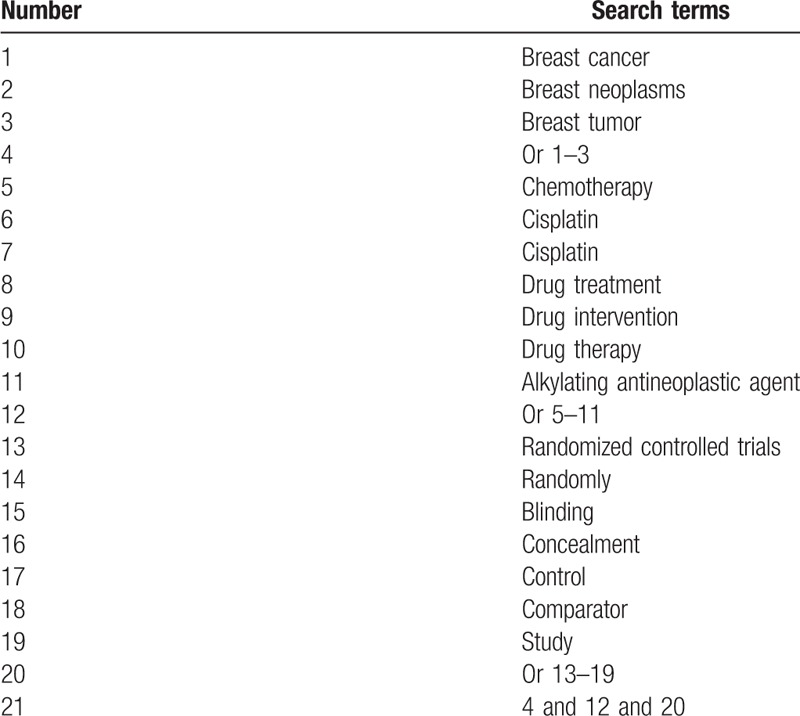
Search strategy for PubMed.

### 2.4 Data collection and management

#### 2.4.1 Study selection

Two authors will independently scan all titles and abstracts identified from the search strategy for all databases. All duplicated and irrelevant studies will be excluded. Any discrepancies between 2 authors will be solved by a third author via discussion. Full reports will be further obtained for all remaining studies to judge whether they meet the final eligibility criteria. The results of study selection will be presented in the flowchart.

#### 2.4.2 Data extraction

Two authors will carry out data extraction using a standardized data extraction table to collect relevant data from each eligible trial. The data comprises of publication details, study characteristics, study setting, study design, sample size, patient characteristics, diagnostic criteria, eligibility criteria, experimental and control details, outcome measurements, safety, and follow-up information. Any divergences regarding data extraction between 2 authors will be solved by a third author via discussion. If there is insufficient or missing information, we will contact corresponding authors of primary RCTs. If we cannot receive those data, we will just analyze available data. Moreover, we will discuss its possible impacts in the text.

### 2.5 Risk of bias assessment for eligible studies

In this study, risk of bias for all eligible studies will be assessed using Cochrane risk of bias tool. This tool has 7 aspects and each aspect is judged as 3 levels: high risk of bias, unclear risk of bias, and low risk of bias. Two authors will independently perform risk of bias for all included studies. All different opinions between 2 authors will be solved by a third author through discussion.

### 2.6 Treatment effect measurements

For continuous outcomes, mean differences or standardized mean differences with 95% confidence intervals will be exerted. For dichotomous outcomes, risk ratios or odds ratios with 95% confidence intervals will be calculated.

### 2.7 Statistical analysis

We will use RevMan V.5.3 software (London, UK) for statistical analysis in this study. We will use *I*^2^ statistic test to identify heterogeneity among eligible studies. Values of *I*^2^ ≤ 50% exert low heterogeneity, and a fixed-effect model will be used. In addition, meta-analysis will be carried out. On the other hand, values of *I*^2^ > 50% mean significant heterogeneity, and a random-effect model to will be applied. Under such situation, we will perform subgroup analysis to explore any factors of such high heterogeneity. We will report outcome results as a narrative summary if substantial heterogeneity still exerts after subgroup analysis.

Subgroup analysis will be performed based on the different treatments, controls, and outcomes. Sensitivity analysis will also be carried out by removing studies with high risk of bias. Finally, we will investigate the reporting bias if sufficient trials are entered using funnel plots and Egger linear regression test.

## 3 Discussion

BC is one of most common cancers in female population. WC has been widely used clinically to treat this disorder. Currently, there is limited evidence to determine whether WC has similar effect on patients with BC. Therefore, the comparisons of the efficacy and safety will be investigated between WC and other interventions in the experimental group and control group. This study will provide high-quality evidence-based medicine to determine whether WC is an effective and safety treatment for patients with BC.

## Author contributions

**Conceptualization:** Ying Ma, Ning An, Yan-cui Liu.

**Data curation:** Ying Ma, Nai-peng Zhang, Wen-yuan Li, Wei Zhao, Yan-cui Liu.

**Formal analysis:** Ying Ma, Nai-peng Zhang, Ning An, Wen-yuan Li, Wei Zhao.

**Funding acquisition:** Yan-cui Liu.

**Investigation:** Yan-cui Liu.

**Methodology:** Nai-peng Zhang, Ning An, Wen-yuan Li, Wei Zhao.

**Project administration:** Ning An, Yan-cui Liu.

**Resources:** Nai-peng Zhang, Ning An, Wen-yuan Li, Wei Zhao, Yan-cui Liu.

**Software:** Ying Ma, Nai-peng Zhang, Ning An, Wen-yuan Li, Wei Zhao.

**Supervision:** Ying Ma, Yan-cui Liu.

**Validation:** Ying Ma, Ning An, Wei Zhao.

**Visualization:** Nai-peng Zhang, Wen-yuan Li, Wei Zhao.

**Writing – original draft:** Ying Ma, Nai-peng Zhang, Ning An, Wen-yuan Li, Wei Zhao, Yan-cui Liu.

**Writing – review & editing:** Ying Ma, Nai-peng Zhang, Ning An, Wen-yuan Li, Wei Zhao, Yan-cui Liu.
